# Glutamine: Metabolism and Immune Function, Supplementation and Clinical Translation

**DOI:** 10.3390/nu10111564

**Published:** 2018-10-23

**Authors:** Vinicius Cruzat, Marcelo Macedo Rogero, Kevin Noel Keane, Rui Curi, Philip Newsholme

**Affiliations:** 1 School of Pharmacy and Biomedical Sciences, Curtin Health Innovation Research Institute, Biosciences, Curtin University, Perth 6102, Australia; Kevin.Keane@curtin.edu.au; 2 Faculty of Health, Torrens University, Melbourne 3065, Australia; 3 Department of Nutrition, Faculty of Public Health, University of São Paulo, Avenida Doutor Arnaldo 715, São Paulo 01246-904, Brazil; mmrogero@usp.br; 4 Interdisciplinary Post-Graduate Program in Health Sciences, Cruzeiro do Sul University, São Paulo 01506-000, Brazil; ruicuri59@gmail.com

**Keywords:** nutrition, amino acids, leukocytes, skeletal muscle, gut, liver

## Abstract

Glutamine is the most abundant and versatile amino acid in the body. In health and disease, the rate of glutamine consumption by immune cells is similar or greater than glucose. For instance, in vitro and in vivo studies have determined that glutamine is an essential nutrient for lymphocyte proliferation and cytokine production, macrophage phagocytic plus secretory activities, and neutrophil bacterial killing. Glutamine release to the circulation and availability is mainly controlled by key metabolic organs, such as the gut, liver, and skeletal muscles. During catabolic/hypercatabolic situations glutamine can become essential for metabolic function, but its availability may be compromised due to the impairment of homeostasis in the inter-tissue metabolism of amino acids. For this reason, glutamine is currently part of clinical nutrition supplementation protocols and/or recommended for immune suppressed individuals. However, in a wide range of catabolic/hypercatabolic situations (e.g., ill/critically ill, post-trauma, sepsis, exhausted athletes), it is currently difficult to determine whether glutamine supplementation (oral/enteral or parenteral) should be recommended based on the amino acid plasma/bloodstream concentration (also known as glutaminemia). Although the beneficial immune-based effects of glutamine supplementation are already established, many questions and evidence for positive in vivo outcomes still remain to be presented. Therefore, this paper provides an integrated review of how glutamine metabolism in key organs is important to cells of the immune system. We also discuss glutamine metabolism and action, and important issues related to the effects of glutamine supplementation in catabolic situations.

## 1. Introduction

At the most basic level, amino acids are the building blocks of proteins in our cells and tissues, and after water are the second most abundant compound in mammals. Amino acids can be obtained from endogenous and/or exogenous (i.e., diet) proteins, and their availability is of fundamental importance for cell survival, maintenance, and proliferation. Mammals, in particular, have developed biochemical and metabolic pathways to control pathogen infection by increasing amino acid catabolism to aid immune responses, thus restricting the availability of nitrogen-containing nutrients to invading microorganisms [1]. This evolutionary mechanism also becomes advantageous for the host to control its own inflammatory responses to infections.

Among the 20 amino acids detailed in the genetic code, glutamine provides the best example of the versatility of amino acid metabolism and immune function. Glutamine is the most abundant and versatile amino acid in the body, and is of fundamental importance to intermediary metabolism, interorgan nitrogen exchange via ammonia (NH_3_) transport between tissues, and pH homeostasis. In almost every cell, glutamine can be used as a substrate for nucleotide synthesis (purines, pyrimidines, and amino sugars), nicotinamide adenine dinucleotide phosphate (NADPH), antioxidants, and many other biosynthetic pathways involved in the maintenance of cellular integrity and function [2,3,4].

Most cells in the body function with a constant turnover and/or supply of nutrients, however, cells of the immune system frequently have to function under nutrient restricted microenvironments [1]. Although glucose is a vital metabolite, and the main fuel for a large number of cells in the body, cells of the immune system, such as lymphocytes, neutrophils, and macrophages, utilize glutamine at high rates similar to or greater than glucose under catabolic conditions, such as sepsis, recovery from burns or surgery, and malnutrition, as well as high intensity/volume physical exercise [5,6]. This theory was first experimentally confirmed in the 1980’s by the laboratory of Eric Newsholme (1935–2011, widely accepted now as the origin of hypotheses and evidence for the concept of “immunometabolism”) [3,7] in the University of Oxford, and subsequently by many other laboratories worldwide [4,8,9]. For this reason, glutamine is considered as a “fuel for the immune system”, where a low blood concentration may impair immune cell function, resulting in poor clinical outcomes and increased risk of mortality [10].

Currently, glutamine is routinely supplied as a component of clinical nutrition supplementation for pre-and post-operative patients, and also for many elite athletes to restore immune functions. Although there is a growing evidence in support of the immune mediating effects of glutamine supplementation, several questions and specific considerations still remain. Therefore, the aim of the present study is to provide an integrated review on how glutamine metabolism in key organs, such as the gut, liver, and skeletal muscles, is important to cells of the immune system. These key organs control glutamine availability and shed light on important considerations in regards to glutaminemia (glutamine concentration into the bloodstream). The immune-enhancing properties and related paradigms of glutamine supplementation in health and disease are also discussed herein.

## 2. A Brief Overview of Glutamine Metabolism

Glutamine is an l-α-amino acid containing five carbons; its molecular weight is 146.15 kDa and its elemental composition comprises carbon (41.09%), hydrogen (6.90%), oxygen (32.84%), and nitrogen (19.17%). With respect to its physiological pH, glutamine is classified as a neutral amino acid, whereas it is nutritionally classified as a non-essential amino acid. Glutamine has two amino groups, namely the α-amino group and the easily-hydrolysable side-chain amide group, and these features enable the role played by glutamine as a nitrogen transporter and NH_3_ carrier. Glutamine is also a proteinogenic amino acid, i.e., amino acids that are incorporated into proteins, and accounts for 5 to 6% of bound amino acids [9].

Healthy individuals weighing about 70 kg have approximately 70 to 80 g of glutamine distributed in the whole body [11]. Using isotopic and pharmacokinetic techniques, it has been estimated that glutamine endogenous production is between 40 to 80 g/day [12,13]. In plasma obtained from blood samples, glutamine concentration varies between ~500 to 800 μM/L (values recorded after 12-h in a fasting state), which represents about 20% of the total free amino acids pool in the blood [9]. In tissues, such as the liver and the skeletal muscles, glutamine concentration is even higher than in plasma, representing about 40% to 60% of the total amino acid pool [14,15]. In both, plasma and tissues, glutamine concentration is 10- to 100-fold in excess of any other amino acid, and for this reason, glutamine is considered the body’s most abundant amino acid.

In the whole body, glutamine concentration and availability depends on the balance between its synthesis and/or release, and uptake by human organs and tissues. The lungs, liver, brain, skeletal muscles, and adipose tissue have organ tissue-specific glutamine synthesis activity. On the other hand, primarily glutamine-consuming tissues, such as the intestinal mucosa, leukocytes, and renal tubule cells, have high glutaminase activity and cofactors capable of degrading glutamine. However, the liver may become a glutamine-consuming site, and tissues, such as muscle tissue, may present reduced glutamine synthesis under certain conditions, such as reduced carbohydrate [16] and/or amino acid [17] intake, high catabolic situations, and/or diseases and stress [5]. Many other factors—mainly glucocorticoids [18], thyroid hormones [19], growth hormone [20], and insulin [21]—can modulate the activity performed by glutamine metabolism-regulating enzymes.

**Figure 1:**
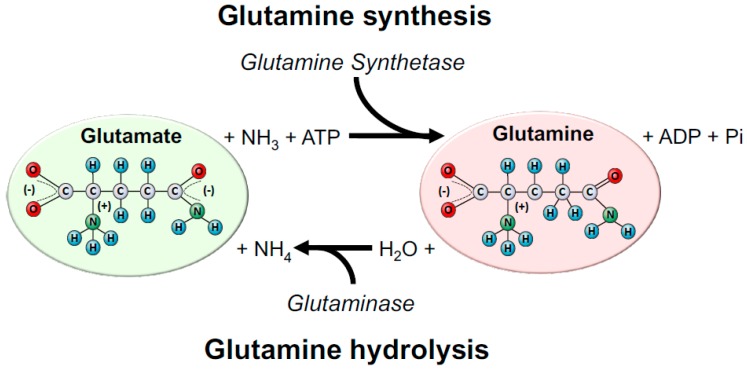
Glutamine synthesis and hydrolysis. Glutamine is mainly synthesized by the enzyme glutamine synthetase (GS) and hydrolysed by the enzyme, glutaminase (GLS). GS catalyses glutamine biosynthesis using glutamate and ammonia (NH_3_) as a source. In this reaction, one ATP is consumed. Glutamate can be donated by many amino acids obtained exogenously (i.e., diet) and/or from endogenous amino acids’ catabolism. On the other hand, GLS is responsible for glutamine hydrolysis to glutamate and ammonium ion (NH_4_). Almost all cells in the body express GS and GLS, and their predominant expression and activity will dictate if the tissue is more likely to produce or consume glutamine in health and disease.

Several enzymes are involved in glutamine metabolism; the two main intracellular enzymes are glutamine synthetase (GS, EC 6.3.1.2) and phosphate-dependent glutaminase (GLS, EC 3.5.1.2). GS is responsible for triggering the reaction that synthesizes glutamine from ammonium ion (NH_4+_), and glutamate through ATP consumption, whereas GLS is responsible for glutamine hydrolysis, which converts it into glutamate and NH_4+_ again [22,23] (Figure 1). With respect to the intracellular location, GS is primarily found in the cytosol, whereas GLS (in its active form) is mainly found within the mitochondria. These locations are compatible with the enzymes’ functions: GS produces glutamine for the synthesis of cytoplasmic proteins and nucleotides, whereas GLS catalyses glutamine conversion to glutamate as an important step to the tricarboxylic acid cycle (TCA, also known as the Krebs cycle) entry at 2-oxoglutarate as an energy source or source of metabolic intermediates [3].

**Figure 2:**
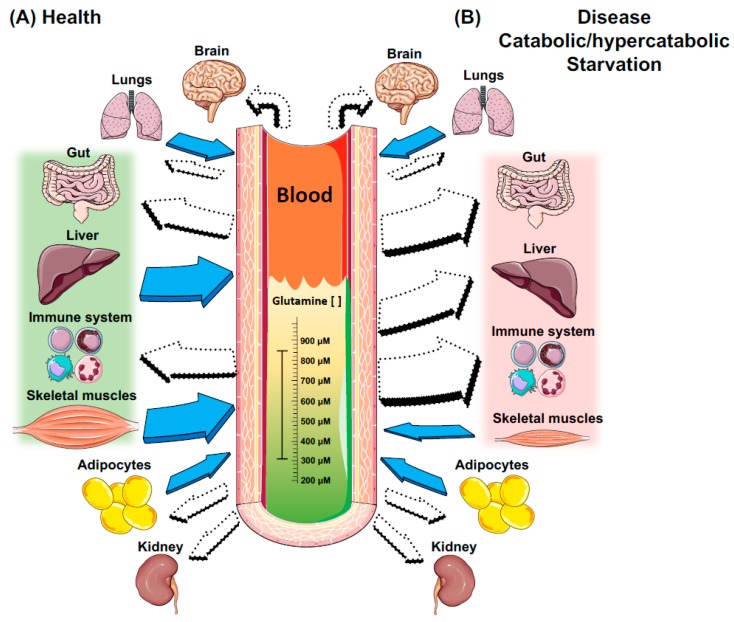
Intertissue glutamine production and utilisation in health and catabolic/hypercatabolic situations. Filled arrows indicate tissues that exhibit GS activity and thus produce glutamine; white arrows indicate tissues that exhibit GLS activity, and thus consume glutamine. In health and/or fed states, glutamine stores are in equilibrium in plasma/bloodstream and tissues, and are maintained constantly mainly by the liver and skeletal muscles, two major stores of glutamine in the body. On the other hand, cells of the immune system are extremely dependent on glucose and glutamine in situation (**A**), and even more in situation (**B**). Although the gut is a major site of glutamine consumption, in situation (**B**), there is a dramatic increase in glutamine consumption from both the luminal and basolateral membrane, when compared to situation (**A**). In addition, the liver switches the role of a major producer to a major glutamine consumer to maintain gluconeogenesis, and the whole body relies on the skeletal muscle’s ability/stores to maintain glutamine levels. However, this process is usually accompanied by a dramatic increase in muscle proteolysis, atrophy, and cachexia. The lungs and adipose tissue exhibit both GS and GLS enzymes, and hence can produce and consume glutamine in situations (**A**) and (**B**). The brain and the kidneys do not exhibit GS, only GLS, and hence are mainly dependent on plasma glutamine availability in situations (**A**) and (**B**).

Glutamine synthesis by the GS depends on glutamate availability. Glutamate, in turn, is synthesized from 2-oxoglutarate NH_4_, through the action of the glutamate dehydrogenase, or even from the catabolism of other amino acids, such as branched-chain amino acids (BCAAs), mainly leucine [17,24]. Studies conducted with rats reported that BCAAs, such as leucine, can be almost exclusively transaminated with α-ketoglutarate to form glutamate, which in turn can incorporate free NH_3_ and under the action of GS form glutamine [6,24] (Figure 2).

**Figure 3:**
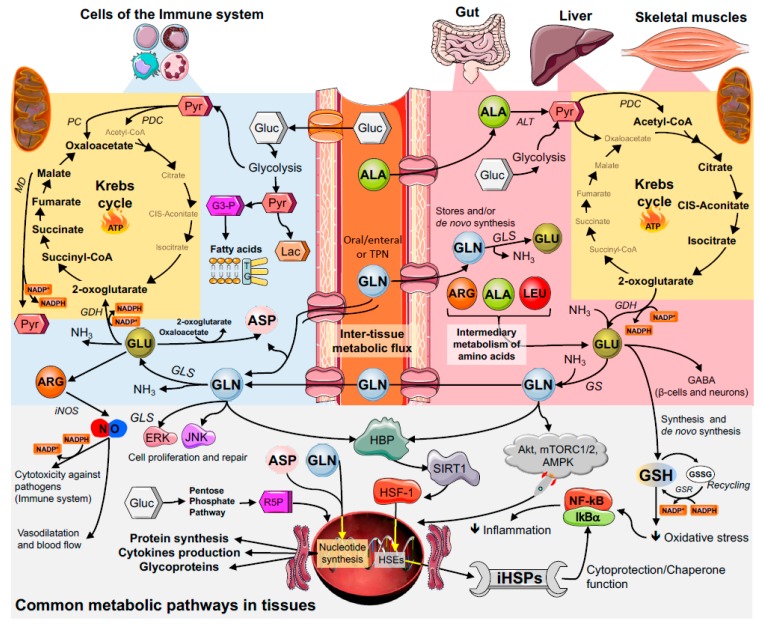
Glutamine inter-tissue metabolic flux starting in skeletal muscle, liver, and gut continues in the immune cells. Abbreviations: Glutamine, GLN; glutamate, GLU; aspartate, ASP; arginine, ARG; leucine, LEU; alanine, ALA; glucose, Gluc; pyruvate, Pyr; pyruvate dehydrogenase; PDC; pyruvate carboxylase, PC; malate dehydrogenase, MD; glyceraldehyde-3-Phosphate, G3-P; lactate, Lac; triacylglycerol, TG; ribose 5-phosphate, R5P; alanine aminotransferase, ALT; glutamate dehydrogenase, GDH; glutamine synthetase, GS; glutaminase, GLS; inducible nitric oxide synthase, iNOS; intracellular heat shock protein, iHSP; heat Shock Factor 1, HSF-1; heat shock elements, HSEs; sirtuin 1, SIRT1; hexosamine biosynthetic pathway, HBP; ammonia, NH_3_; glutathione, GSH; oxidized GSH, GSSG; glutathione S-reductase, GSR; protein kinase B, Akt; AMP-activated protein kinase, AMPK; mTOR complex 1 and 2, mTORC1/2, extracellular signal-regulated kinases, ERK; c-Jun N-terminal kinases, JNK; gamma-Aminobutyric acid, GABA.

Glutamine tissue and blood concentrations are dependent on GS or GLS activities. Endogenous glutamine synthesis does not meet the human body’s demands in catabolic conditions, such as in cancer [25], sepsis [4,26], infections [27,28], surgeries [8], traumas [10], as well as during intense and prolonged physical exercise [29,30]. Glutamine assumes the role of a conditionally essential amino acid in such deficiency conditions by promoting a concomitant increase in GLS expression and inhibiting the GS action [14]. However, it is worth emphasizing that, although plasma glutamine concentration is reduced from its normal concentration (i.e., 500–800 μmol/L) to 300–400 μmol/L, cells depending on this amino acid, such as immune cells, are in fact poorly influenced in terms of proliferation and function [6]. On the other hand, the high tissue catabolism leads to reduced glutamine stock in human tissues, mainly in the muscle and liver (Figure 3). The low glutamine concentration in human tissues affects the whole body since this amino acid provides nitrogen atoms to the synthesis of purines, pyrimidines, and amino sugars [31]. If the high glutamine degradation in these tissues persists, a large number of metabolic pathways and mechanisms that depend on glutamine availability are affected, resulting in immunosuppression. More recently, studies reported that bacterial infections (e.g., *Escherichia coli*) can alter its metabolism and harness glutamine to suppress the effects of acid stress and copper toxicity [32]. Hence, bacterial pathogens can adapt and survive by altering core metabolic pathways important for host-imposed antibacterial strategies.

## 3. Key Metabolic Organs in Glutamine Homeostasis

### 3.1. The Gut

Both the small and large intestines are capable of metabolizing large amounts of glutamine supplied by both the diet and/or the bloodstream [33,34]. Glutamine for the gut is quantitatively more relevant than glucose as an energy substrate. For example, in the enterocytes, the glutamine carbon can be metabolized by two main pathways, namely: (i) By forming delta^1^-pyrroline-5-carboxylate; (ii) or by conversion to alpha-ketoglutarate as an intermediary in the Krebs cycle. The first pathway enables the formation of proline, ornithine, and citrulline from glutamine carbon by using approximately 10% of the amino acid concentration found in the intestine. Another 10–15% of glutamine is incorporated into the tissue protein; the highest proportion of it (approximately 75%) is metabolized in the Krebs cycle for energy production purposes [14,35].

The glutamine hydrolysis into glutamate, which is catalysed by GLS, corresponds to the first reaction resulting from glutamine consumption [36]. Although the gut is the major site of glutamine consumption, glutamine concentration in the intestinal tissue is low. This is due to the high GLS activity (3–6 μmol/hour/mg of protein), and also high GLS affinity for the substrate, glutamine. Interestingly, there is a correlation between the presence of GSL and the use of glutamine by certain cell types. Almost all GLS found in the intestinal cells is bound to the mitochondrial membrane. The modulation of GLS activity in the intestinal tissue is important to maintain the tissue integrity and enable adequate absorption of nutrients, as well as to prevent bacterial translocation into the bloodstream (i.e., septicaemia) [37]. Prolonged fasting and malnutrition states are associated with reduced GLS activity; on the other hand, GLS activity increases in the postprandial period, after the administration of enteral feeding of branched-chain amino acids and/or l-alanyl-l-glutamine [38].

The ATP-ubiquitin-dependent proteolytic pathway associated with proteasome is known to degrade endogenous short-lived or abnormal proteins/peptides, as well as participating in the regulation of the inflammatory response. The ATP-ubiquitin-dependent proteolytic pathway could be important for the turnover of gut mucosal proteins, which are very short lived. Indeed, the nuclear factor of kappa light polypeptide gene enhancer in B-cells inhibitor (IκB) ubiquitinylation allows the nuclear factor kappa-light-chain-enhancer of activated B cells’ (NF-κB) translocation in the nucleus, and thus transcription of proinflammatory genes [17,39,40] (Figure 3). Glutamine stimulates protein synthesis and reduces ubiquitin-dependent proteolysis in the enterocyte since this amino acid reduces ubiquitin gene expression. Glutamine can increase the gene expression of the arginine-succinate synthase enzyme in Caco-2 cells (human colon epithelial-line cell). Glutamine activates the extracellular signal-regulated kinases (ERKs) and the c-Jun N-terminal kinases (JNK) in the enterocyte, and it leads to significant increase in c-Jun gene expression and in the activity of the transcription factor known as activator protein 1 (AP-1) [41,42]. Such glutamine action potentiates the effects of growth factors on cell proliferation and repair. Heat shock (43 °C) induces intestinal epithelial cell death, which can be exacerbated due to the lack of glutamine. However, as it happens with muscle tissues, glutamine supplementation enables a dose-dependent reduction in heat shock-associated cell death. This effect of glutamine partly results from the amino acid capacity of increasing the gene expression of HSP 70 [37].

Dysregulation of cytokine production plays a major role in the pathogenesis of inflammatory bowel disease (IBD). The gut mucosa of patients with IBD (Crohn’s disease or ulcerative colitis) has been reported to produce high amounts of proinflammatory cytokines, such as interleukin (IL-)1β, IL-6, IL-8, and tumour necrosis factor-alpha (TNF-α), in contrast to a less marked increase in the production of anti-inflammatory cytokines, such as IL-10. For example, Coeffier, et al. [43] verified that glutamine reduces pro-inflammatory cytokine production by human intestinal mucosa, probably by a posttranscriptional pathway. Glutamine could be useful to modulate inflammatory conditions with imbalanced cytokine production.

### 3.2. Skeletal Muscles

The body’s glutamine availability and metabolism are directly associated with the skeletal muscle tissue. Skeletal muscles are quantitatively the most relevant site of glutamine stock, synthesis, and release despite the relatively-low GS enzyme activity per muscle tissue-unit mass [11]. Thus, skeletal muscles play a fundamental role in glutamine metabolism, since it is one of the most abundant tissues found in the human body [44]. The intramuscular glutamine content corresponds to 50–60% of the total free amino acids found in the skeletal muscle tissue. Approximately 80% of the body glutamine is found in the skeletal muscle, and this concentration is 30 times higher than that recorded for human plasma [45,46]. The free amino acid concentrations in the muscle tissue depend on the muscle fiber type. Studies conducted in the skeletal muscle of rats showed that glutamine concentration was three times higher in slow-twitch muscle fibers (type 1 fibers) than in fast-twitch muscle fibers (type 2 fibers). High glutamine concentration in slow-twitch muscle fibers is due to the high GS enzyme activity and ATP availability for glutamine synthesis [47].

Hormones, such as insulin and insulin-like growth factors (IGFs), stimulate glutamine transport into the intracellular environment, whereas glucocorticoids stimulate glutamine release into the extracellular space. The transmembrane gradient for glutamine through the skeletal muscle is high, a fact that restricts amino acid diffusion through the cell membrane. Glutamine is actively transported into cells through a sodium-dependent channel system, whose outcome is a consumption of ATP. The glutamine transport through the muscle cell membrane is faster than the transport of all other amino acids [48]. Interestingly, the constant maintenance of glutamine availability in the intracellular fluid, as well as the high glutamine concentration gradient across the cell membrane, is supported by many pathways, such as the transport system affinity for the amino acid, its intracellular turnover ratio and the extracellular supply, the intra- and extracellular sodium concentrations, and the influence of other amino acids competing for carrier molecules [49,50].

During the post-absorptive state, approximately 50% of the glutamine synthesis in the skeletal muscle takes place through glutamate uptake from the bloodstream, a fact that characterizes part of the glutamine-glutamate cycle. In addition, muscle protein catabolism directly produces glutamine, although it also leads to BCAAs, glutamate, aspartate, and asparagine release. The carbon skeletons of these amino acids are used for glutamine de novo synthesis [51,52]. Glutamine and alanine correspond, respectively, to 48% and 32% of the amino acids released by the skeletal muscle in the post-absorptive state; the glutamine containing two nitrogen atoms per molecule is the main muscle nitrogen-release source. The glutamine and alanine exchange rates exceed their abundance in the body, and their occurrence in the muscle protein corresponds to 10–15%, thus indicating the constant need of glutamine and alanine de novo synthesis in the skeletal muscle [4]. The glutamine synthesis rate in the skeletal muscle (approximately 50 mmol/h) is higher than that recorded for any other amino acid. Thus, glutamine and alanine should result from the interconversion of amino acids within the cell, in a process that depends on the cell metabolic conditions, which are affected by human nutritional and hormonal status, as well as by physical exercise [53,54].

One of the first studies about muscle glutamine metabolism in catabolic situations has recorded that reduced glutamine concentration in the skeletal muscle is associated with the reduced survival rate of sepsis-state patients. The severe muscle glutamine-concentration decrease in critically-ill patients (80% reduction, on average, in the normal concentration due to protein degradation) is accompanied by increased glutamine synthesis and release from the skeletal muscle. It happens because of the increased messenger RNA (mRNA) and GS enzyme activity in the skeletal muscle during severe catabolic states. Glucocorticoids may increase the amount of GS mRNA in the muscle tissue through a glucocorticoid receptor-dependent process that happens in the cytosol. Once the glucocorticoid is bound to its cytosolic receptor they are translocated to the nucleus, where they bind to regions containing glucocorticoid-response elements, which induce GS gene transcription, among others [55,56].

Although the GS activity increases in response to physiological stress, the protein amount may not increase in parallel to that of the mRNA, thus indicating the activation of post-transcriptional control mechanisms. Thus, the activity of the aforementioned enzyme appears to be controlled through the intracellular glutamine concentration by means of a post-transcriptional control mechanism, which increases the GS enzyme activity when the intracellular glutamine concentration decreases. However, the GS enzyme is relatively unstable in the presence of glutamine; therefore, the increased intracellular glutamine concentration leads to faster GS degradation. In addition, glucocorticoids and intracellular glutamine depletion work synergistically by increasing the GS expression in the skeletal muscle [57].

In vitro studies conducted with several cell types demonstrated that glutamine can also change the gene expression of contractile proteins. According to one study, cardiomyocyte growth and maturation were accompanied by increased mRNA contents in proteins, such as alpha-myosin heavy-chain (α-MHC) and alpha-actin; both parameters were considered non-pathologic hypertrophy [58]. Other studies highlight the relevant role played by glutamine in mediating the activation of pathways, such as the mammalian target of rapamycin (mTOR), which is considered an essential tissue size and mass regulator, either in healthy or ill patients. In fact, the use of amino acids, mainly of leucine, as anabolic inducers in muscle cells has its action compromised via mTOR when glutamine is not available [17]. Despite the essential role played by glutamine in regulating the expression of muscle content-associated genes, there are no in vivo studies supporting the hypothesis that supplementation applied alone can promote muscle mass increase.

Another significant role played by glutamine is associated with its capacity of modulating protective and resistance responses to injuries, which are also known as antioxidant and cytoprotective effects. The high oxidative stress generated in catabolism situations results in several effects that culminate in pro-apoptotic stimuli through classic pathways, such as that of the NF-κB. Reactive oxygen species (ROS), both the radical and the non-radical species, react to minerals, to phospholipid membranes, and to proteins, among other relevant compounds, to cellular homeostasis [59]. Glutamine can modulate the expression of heat shock proteins (HSP). According to a study conducted with acutely-inflamed mice (subjected to endotoxemia, which is a sepsis model), the increased glutamine availability in the animals’ tissues helped to keep the HSP expression, mainly in the 70 (the most abundant form), 90, and 27 kDa family. Results concerning skeletal and liver muscles were recorded at protein and gene expression levels. In addition, other genes were highly responsive to glutamine, such as the heat shock factor 1 (HSF-1), which is important for HSP synthesis, and enzymes linked to the antioxidant system (Figure 3). The glutamate resulting from glutamine is an essential substrate for glutathione synthesis, a fact that changes the expression of genes, such as glutathione S-reductase (GSR) and glutathione peroxidase 1 (GPx1). The glutamine cytoprotective and antioxidant properties may be particularly important in high catabolism situations, in which the activity and the expression of inflammatory pathways mediated by NF-κB are modulated [4,39].

### 3.3. The Liver

The liver is highly metabolic and has many functions, including the detoxification of blood constituents arriving from the digestive tract, production of bile to aid digestion, metabolism of carbohydrates, lipids, proteins, and drugs, blood pH balance, synthesis of plasma proteins, and the storage and synthesis of glycogen and lipids. As indicated already, glutamine is an important precursor for the generation of other metabolites, such as amino acids (glutamate), TCA components (α-ketoglutarate), and nucleotides (AMP, purines, and pyrimidines), along with the activation of the chaperone function (mediated by HSP response) and antioxidant defence (mediated by glutathione, GSH). Therefore, glutamine is crucial for energy metabolism and proliferation of hepatocytes in the liver. In addition, glutamine is an important precursor for gluconeogenesis, the process of glucose production from other non-carbohydrate constituents, which is a central metabolic pathway in the liver that allows maintenance of blood glucose levels in fasting and starvation conditions following depletion of glycogen stores. During severe illness, the skeletal muscle is the major supplier of glutamine, while the liver is a major consumer [28,60]. This consumption supports many of the liver activities listed above, but as a key amino acid involved in nitrogen metabolism, the uptake of glutamine in liver hepatocytes regulates the activity of the urea cycle due to its conversion to glutamate NH_3_ by GLS [61]. Consequently, the liver regulates blood pH and the detoxification of NH_3_ via the urea cycle using glutamine.

NH_3_ and NH_4_^+^ are toxic metabolic remnants of amino acid catabolism, and blood delivered to the liver from the gut is rich in NH_3_/NH_4_^+^. In hepatocyte mitochondria, the reaction of NH_3_ with ATP and the bicarbonate ion (HCO_3_^−^) leads to the formation of carbamoyl phosphate (CP) by CP synthetase (CPS), a key intermediate in the urea cycle. Under the action of ornithine transcarbamylase, ornithine reacts with CP to form citrulline. This subsequently undergoes various biochemical reactions forming arginine, fumarate, and, ultimately, urea, which is excreted from the hepatocyte cytosol into the blood. In the final urea-forming reaction, ornithine is regenerated to allow the cyclic continuation of the urea cycle with additional NH_3_. Importantly, glutamine concentrations in the mitochondria regulate flux through CP generation. High glutamine levels increase glutamate and NH_3_ through GLS activity, with the NH_3_ reacting with ornithine to form CP. NH_3_ is also a co-activator of GLS in the liver [62,63], and since liver GLS is not inhibited by glutamate [61,64], the glutamate produced can form N-acetylglutamate, which is an activator of both GLS and CPS [65,66]. Consequently, both of these biochemical mechanisms result in a high flux toward urea formation due to the elevated levels of NH_3_ in the mitochondrial (sourced from digestive tract blood and glutamine degradation), the high affinity of CPS for NH_3_, and the increased activity of GLS. However, the ultimate generation of urea appears to be regulated by the sub-cellular level of glutamine and, consequently, its uptake from the extracellular environment.

The intra-, inter-, and extracellular transport of glutamine in hepatocytes is central to the conversion of the NH_3_/NH_4_^+^ to urea, its subsequent excretion, and blood pH balance due to effects on HCO_3_^−^. The liver architecture is exquisitely designed such that periportal hepatocytes (near the portal vein) receiving blood and nutrients from the gut are primarily responsible for urea production using glutamine as outlined above. However, the distal hepatocytes near the hepatic vein (perivenous), utilise any remaining NH_4_^+^ from the blood, including that bypassing the periportal hepatocytes, for the re-synthesis and secretion of glutamine into the circulation. In this latter scenario, the NH_3_/NH_4_^+^ enters the perivenous hepatocytes and, using glutamate as a substrate, glutamine is generated by GS. This process scavenges any NH_3_/NH_4_^+^ escaping the periportal process, while also replenishing the glutamine that was used by periportal hepatocytes in the generation of urea and disposal of nitrogen. The different functional regions of the liver were illustrated by the expression status of glutamine-metabolizing enzymes, such that high levels of GLS were found in the portal region [67,68], while only 7% of hepatocytes expressed GS, and these were found specifically around the central hepatic veins [69].

The intercellular or liver compartmentalized cycling of glutamine between these liver regions is also mediated by specific membrane transporters in periportal and perivenous hepatocytes. These transport systems also control intra- and extracellular pH by the antiport translocation of H^+^ on the entry of Na^+^ and glutamine. Glutamine from the diet is taken up by periportal hepatocytes along with two Na^+^ ions, while one H^+^ is extruded in the opposite direction from the hepatocyte into the extracellular space. This process is driven by the concentrations of glutamine and Na^+^ outside the cell (i.e., in the blood), and also the intracellular concentration of H^+^. In essence, this directional transport is also regulated by the relative pH difference between the intracellular and extracellular space, such that periportal hepatic glutamine uptake leads to extracellular acidification and intracellular alkalization, while glutamine export from perivenous hepatocytes leads to intracellular acidification and extracellular alkalization [61]. Experiments in perfused rat livers have shown that a slight alkali increase in extracellular pH (0.4 units) can enhance mitochondrial import of glutamine into hepatocytes, as H^+^ is transported externally to possibly reduce extracellular pH and maintain equilibrium. Mitochondrial glutamine concentration increased to about 15–50 mM, while the extracellular (0.6 mM) and cytosolic (6 mM) glutamine concentrations remain unchanged in these perfusion models [70,71]. Therefore, the regulation and flux through GLS in periportal hepatocytes are regulated by the sub-cellular concentration of glutamine, and not just the rate of glutamine entry from extracellular sources. For perivenous hepatocytes, the synthesis and release of glutamine to the blood is facilitated by the increased cytoplasmic, and lower extracellular concentration of glutamine, along with the less acidic intracellular environment, which is countered by the antiport cytosolic influx of H^+^ [61].

Importantly, glutamine importation and exportation also affect osmotic balance and therefore influences hepatocyte volume. This has additional consequences for hepatic function, including bile synthesis and release [72], but also regulates anabolic processes, such as glycogen, lipid, and protein synthesis [61]. Largely, glutamine uptake enhances hepatocyte cell swelling and hydration [73], which leads to increased glycogen and fatty acid synthesis [74,75], and reduced proteolysis mediated by P38 mitogen-activated protein kinases (p38 MAPK) signalling [76]. Other amino acids, such as glycine and alanine, along with anabolic hormones, such as insulin, promoted hepatocyte swelling, leading to increased biosynthetic processes [77], while catabolic hormones, like glucagon, reduced intracellular glutamine levels induced hepatocyte shrinkage [78]. Consequently, dehydration due to reduced intracellular glutamine levels is characterized by decreased cell volume, initiation of the catabolic process, and insulin-resistant conditions, and it was recently shown that hypertonic infusion can cause glucose dysregulation in humans [79].

The liver is an insulin-sensitive organ and like skeletal muscle, is responsible for glucose disposal via glycogen synthesis. Development of insulin resistance and subsequent glucotoxic conditions can progress to chronic disorders, such as non-alcoholic fatty liver disease (NAFLD), characterized by excessive lipid accumulation, and non-alcoholic steatohepatitis (NASH), characterized by increased extracellular matrix (ECM) deposition [80,81]. These chronic disorders may lead to further hepatocyte damage, manifesting as liver cirrhosis and possibly hepatocellular carcinoma. The liver can be damaged in various ways, including infection (e.g., hepatitis B and C), alcoholism, metabolic disease, and prolonged unhealthy diets. This damage elicits a pro-inflammatory hepatic environment, which leads to liver tissue fibrosis, causing impaired hepatic function [80]. Untreated fibrosis ultimately progresses to cirrhosis, which is mostly irreversible [80]. A key mediator of liver fibrosis is the hepatic stellate cell (HSC), which is a mesenchymal, fibrogenic cell that resides in the sub-endothelial space of Disse between the hepatocyte epithelium and the sinusoids. While normally in a quiescent state, these cells become activated following liver insult, and they respond to cytokines and proliferate to aid injury repair. However, overactivation or a failure to resolve their activation status (chronic activation from continued exposure to pro-inflammatory stimuli) can lead to the increased ECM deposition in the space of Disse that has a negative consequence for hepatocyte function and normal liver architecture, such as loss of microvilli [80,82]. Kupffer cells, a liver macrophage, are also activated in these conditions, and together with HSCs promote a pro-inflammatory hepatic environment. Pro-inflammatory activators of these cells are beyond the scope of this manuscript, but it has been demonstrated that HSCs require glutamine metabolism to maintain proliferation. It was shown that activated HSC were dependent on glutamine conversion to ∝-ketoglutarate and non-essential amino acids for proliferation, and reduction of glutamine significantly impaired HSC activation [82]. In addition, glutamine can be used as a precursor for proline synthesis, which is a key component of collagen and ECM formation [82].

At present, there is some evidence to indicate that glutamine supplements slow NAFLD [83] or NASH [84] progression, but most studies have been conducted in rodents. There is no convincing evidence to indicate that glutamine supplements prevent NAFLD or NASH progression in humans, which may be due to the complexity of multiple factors contributing to these disorders. The liver is a remarkable organ and has the ability to regenerate, and some research has indicated that glutamine supplementation may be advantageous for liver growth and repair after resection, but are again limited to animal studies [85]. However, others have suggested that raised glutamine levels are associated with liver failure, and the severity correlated with plasma glutamine [86]. Consequently, the effects of glutamine on liver function beyond urea synthesis have not been fully explored, and the administration of exogenous glutamine to those with compromised hepatic function needs to be considered carefully [86].

## 4. Glutamine and Immune Cell Function

Glutamine was first considered a biologically important molecule in 1873 when indirect evidence helped to characterize it as a structural component of proteins; then, in 1883, abundant free glutamine was found in certain plants. Interestingly, the number of studies only increased after the research conducted by Sir Hans Adolf Krebs (1900–1981) in the 1930s. At that time, and for the first time in science history, Sir Krebs found that mammalian tissues can hydrolyse and synthesize glutamine [22]. In the 1950s, Eagle, et al. [87] reported that glutamine was utilized by isolated fibroblasts in quantities greater than any other amino acid in the cell incubation medium. Further work at that stage was hampered because glutamine was classified as a non-essential amino acid and it was difficult to measure the levels in plasma and tissues. Throughout the 1960s, 1970s, and 1980s, Hans Krebs, Philip Randle (1926–2006), Derek Williamson (1929–1998), and Eric Newsholme (1935–2011) all worked on metabolic regulation utilizing different research models, from isolated cells in vitro, to human and in vivo experiments. Although glucose is a vital metabolite, and the main fuel for a large number of cells in the body, in the early/mid-1980s, Eric Newsholme was able to advance evidence that glutamine was an important modulator of leukocyte function, such as in lymphocytes [7] and macrophages [88]. One of the authors of this review, Newsholme P et al. (1986; 1987) [88,89,90], reported for the first time that macrophages utilize glutamine actively. Pithon-Curi et al., in 1997 [91,92] described for the first time the consumption of glutamine by neutrophils. The studies by Eric and Philip Newsholme on glutamine metabolism in lymphocytes and macrophages, respectively, prompted many other publications, which jumped from an average of two or three publications per year in the late 1960s and early 1970s to about 50 publications per year in the last 20 years.

During infection and/or high catabolism, the rate of glutamine consumption by all immune cells is similar or greater than glucose [89,90]. However, the increased demand for glutamine by immune system cells, along with the increased use of this amino acid by other tissues, such as the liver, may lead to a glutamine deficit in the human body. In addition, one of the most important sites of glutamine synthesis, the skeletal muscles, reduce their contribution to maintaining plasma glutamine concentration (Figure 2). This effect, depending on the situation may significantly contribute to worsening diseases and infections, and/or increase the risk of subsequent infection, with possible life-threatening implications [93].

In immune cells, glucose is mainly converted into lactate (glycolysis), whereas glutamine is converted into glutamate, aspartate, and alanine by undergoing partial oxidation to CO_2_, in a process called glutaminolysis [3] (Figure 3). This unique conversion plays a key role in the effective functioning of immune system cells. Furthermore, through the pentose phosphate pathway, a metabolic pathway parallel to the glycolysis pathway, cells can produce ribose-5-phosphate (a five-carbon sugar), which is a precursor for the pentose sugars seen in the RNA and DNA structure, as well as glycerol-3-phosphate for phospholipid synthesis [94]. On the other hand, the degradation of glutamine, and thus formation of NH_3_, and aspartate leads to the synthesis of purines and pyrimidines of the DNA and RNA. The expression of several genes in immune system cells is largely dependent on glutamine availability [3]. For example, the role glutamine plays in the control of proliferation of immune system cells occurs through activation of proteins, such as ERK and JNK kinases. Both proteins act on the activation of transcription factors, such as JNK and AP-1, and it leads to the transcription of cell proliferation-related genes. For instance, appropriate glutamine concentration leads to the expression of key lymphocyte cells surface markers, such as CD25, CD45RO, and CD71, and the production of cytokines, such as interferon-gamma (IFN-γ), TNF-α, and IL-6 [2,31,95,96]. Thus, glutamine acts as an energy substrate for leukocytes and plays an essential role in cell proliferation, tissue repair process activity, and intracellular pathways associated with pathogen recognition [97].

### 4.1. Neutrophils

The primary substrate for neutrophil survival endocytosis and ROS generation is glucose. However, glucose is not the only energy metabolite source by these cells. Interestingly, when compared to other leukocytes, such as macrophages and lymphocytes, neutrophils consume glutamine at the highest rates [98,99]. Much of the glutamine is converted to glutamate, aspartate (via Krebs cycle activity), and lactate in neutrophils. Under appropriate conditions, CO_2_, glutamine, and glutamate play an important role in the generation of essential compounds for leukocytes’ metabolism and function, including GSH. Neutrophils use protein structures composed of uncondensed chromatin and of antimicrobial factors also called neutrophilic extracellular traps (NETs). The action of NETs requires ROS formation, synthesis of enzymes, such as myeloperoxidase (MPO) and elastase, as well as components capable of overriding virulence factors and destroying extracellular bacteria [100]. The process involving ROS depends on the activation of the NADPH oxidase 2 (NOX2) complex. Based on glutamine, the malate synthesis uses malic enzyme to produce substantial amounts of NADPH, since it is necessary to form the superoxide anion (O_2_^−^), which presents antimicrobial activity. Similarly, macrophages use glutamine for arginine and thus nitric oxide (NO) synthesis through the action of the inducible NO synthase (iNOS) enzyme, by using NADPH as an energy source. Glutamine increases superoxide generation through NADPH oxidase in neutrophils. 6-Diazo-5-oxo-l-norleucine (DON), an inhibitor of phosphate-dependent glutaminase and thus of glutamine metabolism, causes a significant decrease in superoxide production by neutrophils stimulated with phorbol myristate acetate (PMA). PMA raises mRNA’s expression of gp91, p22, and p47, major components of the NADPH oxidase complex. Glutamine increases expressions of these three proteins either in the absence or in the presence of PMA. Glutamine enhances superoxide production in neutrophils, probably via the generation of ATP and regulation of the expression of components of the NADPH oxidase complex [101]. Glutamine plays a role to prevent the changes in NADPH oxidase activity and superoxide production induced by adrenaline in neutrophils [102].

### 4.2. Macrophages

Metabolism of glucose and glutamine is profoundly affected during the macrophage activation process [103,104]. The effects of thioglycollate (an inflammatory stimulus) and Bacillus Calmette-Guérin—BCG (an activation stimulus) on macrophage glucose and glutamine metabolism have been studied [105]. Either thioglycollate or BCG enhances activities of hexokinase and citrate synthase, and also glucose oxidation whereas BCG markedly increases glutamine metabolism. Lipopolysaccharide (LPS) administration also causes pronounced changes in macrophage metabolism and function (for a review, see Nagy and Haschemi [106]. Glucose and glutamine metabolism is also involved in polarizing signals that up-regulate the transcriptional programs required in the macrophage capacity to perform specialized functions. Protein kinase B (PKB or Akt), mTOR complex 1 (mTORC1), mTORC2, and AMP-activated protein kinase (AMPK) play a critical role in metabolic pathways and associated signalling activation [107,108]. For instance, extracellular glutamine may function as the specific starvation-induced nutrient signal to regulate mTORC1. [17]. Synthesis and secretion of pro-inflammatory cytokines, such as TNF-α, IL-1, and IL-6, by macrophages are also regulated by glutamine availability.

Different populations of macrophages have now been identified, such as M1 and M2 [109,110,111]. The M1 and M2 are in fact two extremes of a still not completely known spectrum of macrophage activation states [109,111,112]. Reprogramming signalling pathways are involved in the formation of M1 or M2 phenotype macrophages. The metabolic reprogramming of macrophages include key changes in glutamine and glucose metabolism [113]. No reports identified the requirement of fatty acids for human macrophage IL-4 induced polarization [114]. This issue, however, remains controversial. Interestingly, macrophages reprogram their metabolism and function to polarize for pro- or anti-inflammatory cells, and this is a consequence of the environmental conditions and stimuli [115]. Treatment of macrophages with LPS promotes a switch from glucose-dependent oxidative phosphorylation to aerobic glycolysis—the Warburg effect [116]. Pyruvate kinase M2 regulates the hypoxia-inducible factor 1-alpha (Hif-1α) activity and IL-1β expression, being a key molecule to induce the Warburg effect in LPS-activated macrophages [117]. Due to this mechanism, M1 macrophages exhibit a quick increase in ATP formation that is required for the host defence response [113,118,119]. The TCA cycle of M2 macrophages has no metabolic flux escape whereas M1 macrophages (treated with LPS) have two points of substrate flux deviation, one occurring at the isocitrate dehydrogenase step reaction and another one at post succinate formation. As a result, there is an accumulation of TCA cycle intermediates (e.g., succinate, α-ketoglutarate, citrate, and itaconate) that regulates LPS macrophage activation [119]. Itaconate has anti-inflammatory properties through activation of nuclear factor erythroid 2-related factor 2 (Nrf2) via Kelch-like ECH-associated protein 1 (KEAP1) alkylation [97]. The glutamine seems to be fully required for IL-4 induction of macrophage alternative activation [120,121]. Liu, et al. [122] reported α-ketoglutarate, generated through glutaminolysis, promotes M2 macrophage differentiation. PPARγ has been reported to be required for IL-4 induced gene expression and stimulation of macrophage respiration and glutamine oxidation [123]. Macrophage metabolism feature varies with the specific-tissue microenvironment, and this is of critical importance for the tissue-resident macrophage function. The peritoneum is rich in glutamate, a product of glutamine metabolism that is used by resident macrophage to induce specific metabolic changes under microbial sensing [121]. Taken as a whole, glutamine metabolism does play a very important role as a synergistic supporter and modulator of macrophage activation.

### 4.3. Lymphocytes

Lymphocyte activation is associated with specific metabolic pathways to optimize its function. The integration of multiple extracellular signals affects transcriptional programs and signalling pathways that determine, in CD4+ T cells, for example, multiple events that include modulation of energy metabolism, cell proliferation, and cytokine production. Associated bioenergetic processes are dependent on the activation of AMPK, indicating cross-talk between metabolism and signalling pathways in immune cell differentiation. Greiner, et al. [124] reported in rat thymocytes that the use of the anaerobic glycolytic pathway is strongly increased after antigenic stimulation with Concanavalin (ConA). Eric Newsholme’s group was the first to report the utilization of glutamine by lymphocytes [125]. Glutamine plays an important role for the function of these cells in different ways. Pyruvate is a common product of glucose and glutamine metabolism in the cells. Curi, et al. [126] described that mesenteric lymphocytes have increased pyruvate oxidation through pyruvate carboxylase when stimulated with ConA, indicating that both glucose and glutamine are involved in the control of lymphocyte proliferation and function. Mitochondria have been reported to be able to regulate leukocyte activation. Succinate, fumarate, and citrate, metabolites of the Krebs cycle and produced through glucose and glutamine metabolism, participate in the control of immunity and inflammation either in innate and adaptive immune cells [97].

Most glucose molecules are transported via glucose transporter 1 (GLUT1), which is not observed in non-activated lymphocytes [127]. GLUT1 is an important metabolic marker of lymphocyte activation as it migrates rapidly to the cell surface after stimulation. Glucose deprivation causes a lower rate of basal proliferation, as well as increased production of IL-2, TNF-α, INF-γ, and IL-4 by CD4+ T cells [128]. Activation of intracellular signalling by Akt beyond GLUT1 protein levels further increases glucose uptake and T cell activation. mTOR and AMPK play important and distinct roles in metabolism and immunity. The stimulation of lymphoid cells leads to increased GLUT1 uptake of glucose by acting on mTOR protein [129]. This pathway is also involved in the differentiation of CD4+ T-cell subsets since mTOR-deficient mice have a decrease in differentiation for effector T lymphocytes [130,131]. In contrast, the AMPK pathway inhibits mTOR by suppressing the signalling of this protein and promotes activation of mitochondrial oxidative metabolism rather than the glycolytic pathway [132,133]. Glutamine is required for T and B-lymphocytes’ proliferation process, as well as for protein synthesis, IL-2 production, and antibody synthesis rates presented by these cells. The evidence has now accumulated that glutamine metabolism plays a key role in the activation of lymphocytes. Glutamine is required for human B lymphocyte differentiation to plasma cell and to lymphoblastic transformation [134].

The cell proliferation process requires both ATP for high-energy expenditure and precursors for the biosynthesis of complex molecules, such as lipids (cholesterol and triglycerides) and nucleotides for RNA and DNA synthesis. To perform rapid proliferation activity under the certain stimulus, lymphocytes switch from oxidative phosphorylation to aerobic glycolysis plus glutaminolysis, and so markedly increase glucose and glutamine utilization. The metabolic transition in a Th0 lymphocyte is crucial for the activation of T cells, since glucose metabolism provides intermediates for the biosynthetic pathways, being a prerequisite for the growth and differentiation of T cells [135]. Glycolysis plays an important role in effector T cell functions associated with the production of inflammatory cytokines, mainly INF-γ and IL-2 [136]. The blockade of glyceraldehyde 3-phosphate dehydrogenase (GAPDH) mRNA by the use of siRNA promotes a reduction of INF-γ in lymphocytes [136]. Therefore, the high glycolytic activity is closely associated with the differentiation of Th0 to Th1 cells [132]. Inhibition of the glycolytic pathway blocks this process whereas it promotes differentiation into Treg cells. Increased glycolysis by proliferating cells is linked to increased uptake of glucose and increased expression and activity of glycolytic enzymes, whereas glucose utilization in the oxidative phosphorylation pathway (OXPHOS) is decreased. Therefore, the “metabolic switch” meets the higher energy requirements, generates metabolic intermediates required for the biosynthesis of macromolecules, and suppresses the metabolic features of rest lymphocytes. Inadequate nutrient delivery or specific metabolic inhibition prevents the activation and proliferation of T cells since the inability to use glucose inhibits T cell differentiation in vitro and in vivo [135,137]. Mitochondrial dynamics are closely associated with T lymphocyte metabolism and function. Activated effector T cells have punctate mitochondria and augmented the activity of anabolic pathways whereas memory T lymphocytes exhibit fused mitochondria and enhanced oxidative phosphorylation activity [138]. HIF1-α plays a central role in the maturation of dendritic cells and the activation of T cells. This factor controls leukocyte metabolism reprogramming, through changes in gene expression, and thus immune cell functions [139].

Glycolysis and glutaminolysis are strongly associated to ensure appropriateness for lymphocyte function. Hexosamine biosynthesis requires glucose and glutamine for the de novo synthesis of uridine diphosphate *N*-acetylglucosamine (UDP-GlcNAc). This sugar-nucleotide inhibits receptor endocytosis and signalling through promoting N-acetylglucosamine branching of Asn (N)-linked glycans. Araujo, et al. [140] reported that high aerobic glycolysis and glutaminolysis activities in a co-operative way decrease the UDP-GlcNAc synthesis and N-glycan branching in mouse T cell blasts due to the low availability of these metabolites for hexosamine synthesis. As a consequence, growth and pro-inflammatory T_H_17 features prevail over anti-inflammatory-induced T regulatory (iTreg) differentiation. The latter process is promoted by IL-2 receptor-α (CD25) loss through endocytosis. The authors then postulated that a primary function of concomitant high aerobic glycolysis and glutaminolysis activities is to limit precursors to N-glycan biosynthesis. This metabolic feature of T lymphocytes has marked implications in autoimmunity and cancer. Glutamine also serves as a precursor for the synthesis of putrescine and the polyamines, spermidine and spermine. High levels of polyamines are reported in tumour cells and in autoreactive B- and T-cells in autoimmune diseases. Polyamines have been described to play a role in the control of normal immune cell function and have been associated with autoimmunity and anti-tumour immune cell properties [141].

## 5. Immunomodulatory Properties of Glutamine Supplementation

Plasma glutamine concentration may be decreased during intense immune cell activity in patients with critical disease conditions; as occurs in sepsis, burn, and injury. Skeletal muscle is the main source of glutamine in mammals. This tissue synthesizes, stores, and releases this amino acid to be used by several organs and cells, such as lymphoid organs and leukocytes [89], as mentioned above. A decrease in plasma/bloodstream glutamine levels results of either insufficient glutamine production in skeletal muscle or excessive consumption by utilizing-cells or both (Figure 2). The decrease in plasma glutamine availability has been reported to contribute to the impaired immune function in several clinical conditions. In fact, glutamine depletion reduces lymphocyte proliferation, impairs expression of surface activation proteins on and production of cytokines, and induces apoptosis in these cells [9]. Addition of glutamine to the diet increases experimental animal survival to a bacterial challenge. Glutamine given through the parenteral route has been reported to be beneficial for patients after surgery, radiation treatment, bone marrow transplantation, or injury [5,142]. Administration of glutamine before the onset of infection prevents it in animals and humans, possibly by preventing deficiency of this amino acid [143].

Concerning the mechanism of action, glutamine regulates the expression of several genes of cell metabolism, signal transduction proteins, cell defence, and repair regulators, and to activate intracellular signalling pathways [2]. Glutamine action also involves signalling pathways’ activation by phosphorylation, such as NF-κB and MAPKs [144]. Thus, the function of glutamine goes beyond that of a metabolic fuel or protein synthesis precursor. This amino acid is also an important regulator of leukocyte function, acting on either gene expression or signalling pathways’ activation.

### 5.1. Glutamine-GSH Axis and the Redox State of the Cell

GSH (γ-l-glutamyl-l-cysteinylglycine) is the most important and concentrated (0.5–10 mmol/L) non-enzymatic antioxidant in mammalian cells. Around 85 to 90% of GSH is found in the cytosol, and about 10 to 15% is located in organelles, such as the mitochondria, nuclear matrix, and peroxisomes. GSH is an antioxidant that can directly react with ROS, generating oxidised GSH (GSSG), and can also donate electrons for peroxide reduction, catalysed by glutathione peroxidase enzyme (GPx) [145]. The redox state of the cell can be obtained from the ratio between the intracellular concentration of glutathione disulphide (GSSG) and GSH, which the ratio is [GSSG]/[GSH], resulting in a reduction of GSH, and an increase in the amounts of GSSG [59]. The redox state of the cells is consequently related to GSH concentrations, which are also influenced by the availability of amino acids. Glutamine (via glutamate), cysteine, and glycine are the precursor amino acids for the synthesis of GSH. However, among these three amino acids, glutamate represents the first and probably the most important step in the synthesis of GSH intermediate compounds. Glutamate synthesis, in turn, is dependent on the glutamine intracellular availability. Thus, a higher glutamine/glutamate ratio reinforces the substrates availability for GSH synthesis [39].

Although all cell types in the human body can synthesize GSH, the liver is quantitatively the main organ for the de novo synthesis of GSH (responsible for ~90% of the circulating GSH in physiological conditions) (Figure 3). The elevated concentration of hepatic GSH is mainly due to the high activity of the enzyme, glutathione reductase, in the γ-glutamyl cycle, also known as Meister’s cycle, in honour to Alton Meister (1922–1995) [146]. This cycle provides GSH to be consumed locally by the liver, or under hormonal regulation (e.g., glucagon, vasopressin, and catecholamines), and GSH can be exported into the plasma and other tissues, such as the skeletal muscles. The intracellular and extracellular GSH concentrations are determined by the balance between its synthesis and degradation, as well as by the ability of the cell to transport GSH between the cytosol and the different organelles or the extracellular space [147].

Free radicals and ROS production are essential for cell signalling and immune-mediated oxidative bursts found in phagocytes, such as neutrophils and macrophages. Conversely, there is growing evidence that chronic and/or exaggerated alterations in the redox balance play an important role in many acute and chronic diseases, such as cancer, cardiovascular disease, diabetes, sepsis, and general infections. For instance, in acute inflammatory situations, such as sepsis or viral infection, there is a rise in the intracellular redox state, and all cell compartments are vulnerable to oxidative stress (characterized by increased levels of ROS, and low removal by the antioxidant system) [10,39]. In this situation, glutamine status becomes even more important in dictating the health/recovery outcome, with low glutamine availability, leading to low antioxidant protection via the glutamine-GSH axis. Indeed, many experimental [4,39,148,149] and observational [10,26] studies have already identified that during high catabolism, the low plasma concentration of glutamine is an independent risk factor for mortality [150].

### 5.2. Heat Shock Protein Response

The ability of all living organisms to respond with rapid and appropriate modifications against physiological challenges is an essential feature for survival. At the most basic cellular level, living organisms respond to unfavourable conditions, such as heat shock, toxins, oxidants, infection, inflammation, and several other stressful situations, by changing the expression of stress-related genes, also known as heat shock genes. This response involves the rapid induction of a specific set of genes encoding for cytoprotective proteins, known as HSP’s [94]. HSP’s are a family of polypeptide proteins clustered according to their molecular weight, which have many intracellular functions. Possibly, the most important function displayed by HSP’s are the action of a molecular chaperone. This function assists protein transport, prevents protein aggregation during folding, and protects newly synthesized polypeptide chains against misfolding and protein denaturation [151]. Although several HSP families have been studied in the last couple of years (e.g., HSP10, HSP25, HSP27; HSP90), the most famous and well described in the literature is the HSP70 (i.e., HSP72 + HSP73) family [29,151,152]. HSP70 acts as anti-inflammatory protein by virtue of turning NF-κB off and attenuating the production of inflammatory mediators [153]. Moreover, HSP70 modulate autophagy by regulating the mTOR/Akt pathway and block signalling pathways associated with protein-degradation [152].

Glutamine found at concentrations similar to those recorded for human plasma leads to a significant HSP72 gene expression increase in peripheral blood mononuclear cells subjected to LPS treatment. On the other hand, reduced glutamine concentration results in reduced HSP72 expression in monocytes; this effect depends on mRNA stability. The preoperative administration of glutamine can modulate HSP70 expression by reducing the activation levels of the cyclic AMP response element binding protein (CREB), which is often associated with exacerbated inflammatory responses. This effect depends on the iNOS activity and leads to an NO production increase. Other studies corroborated the present results and presented similar mechanisms, as well as effects on the expression of other HSP’s, such as HSP25, HSP27, and HSP90.

Glutamine plays a crucial role in the modulation of HSP’s expression through the hexosamine biosynthetic pathway (HBP, Figure 3) [21,94]. In the HBP, glutamine leads to the production of UDP-GlcNAc and (UDP)-N-acetylgalactosamine (UDP-GalNAc) through the enzyme, fructose-6-phosphate amidotransferase (GFAT, the first and rate-limiting step of HBP). UDP-GlcNAc and UDP-GalNAc, in turn, may be attached to serine or threonine hydroxyl moieties in nuclear and cytoplasmic proteins by the enzymic action of O-linked-N-acetylglucosaminyl (O-GlcNAc) transferase (a.k.a. OGT) [94]. The main donors for UDP-GlcNAc are glucose, glutamine, and uridine triphosphate (UTP) from the HBP. Interestingly, both nutrients’ availability and cell stress affect O-GlcNAc downstream signalling, and, not surprisingly, this mechanism is also altered in several metabolic diseases, infection, and inflammatory processes [154,155]. O-GlcNAc synthesis leads to the activation of many transcriptional factors, for instance, Sp1, phosphorylation of Eukaryotic Initiation Factor 2 (eIF2), and sirtuin-1 (SIRT1) [156]. Both Sp1 and eIF2 are key transcription factors for the induction of the main thermal shock eukaryotic factor, HSF-1 [157]. Alternatively, SIRT1 enhances HSF-1 expression and prolongs its activation by binding to the promoters of HS genes, leading to the HSP’s expression [94,158]. Although the O-GlcNAc/Sp1 pathway is considered the main mechanism of the HSP’s gene expression and production, glutamine may also act on HBP via p38/MAPK, leading to the HSP’s expression in cells, such as neutrophils. This response may explain the reduction in neutrophils’ apoptosis after high-intensity physical exercise [144]. Furthermore, by increasing the HBP flux, glutamine stimulates the HSP’s expression by blocking the glycogen synthase kinase 3 beta (GSK-3β), an enzyme that constitutively inhibits HSF-1 activation by phosphorylating the transcription factor at Ser303 [94,159].

In vitro [21,160] and in vivo [4,39,161,162,163] studies demonstrate that glutamine availability maintains cell homeostasis and promotes cell survival against environmental and physiological stress challenges through an enhanced protection mediated by intracellular HSP (iHSP) levels [94]. Interestingly, under severe infection and/or catabolism, low glutamine availability in the body can eventually be accompanied by an aberrant iHSP and lead to the HSP’s release to the extracellular space (eHSP) [4]. eHSP have a wide variety of effects on other cells, including impacting on a cell to cell interaction and chemotaxis, and in some cases, act as a signal to the immune and inflammatory responses. On the other hand, eHSP can also function as a stress signalling and pro-inflammatory molecule by interacting with Toll-like receptors 2 (TLR2) and 4 (TLR4) [164]. This effect can down-regulate iHSP in many cells, leading to apoptosis [4], and has also been associated with increased insulin resistance in skeletal muscle cells [165], and β-cell failure in diabetic individuals. Currently, a novel and overall index of immunoinflammatory status, the extracellular to intracellular HSP70 ratio index (H-index), measured in peripheral blood mononuclear cells (PBMCs) [94], has been established.

## 6. Clinical Translation of Glutamine Delivery

Glutamine is found in relatively high concentrations in vegetable and animal protein-based foods (Table 1). Using a validated food frequency questionnaire (FFQ) of more than 70 thousand participants, Lenders, et al. [166] showed that glutamine (6.85 ± 2.19 g/day), glutamate (7.27 ± 2.44 g/day), and leucine (7.01 ± 2.27 g/day) accounts for the highest intake in protein-based diets. Therefore, a balanced diet provides glutamine, and other essential and non-essential amino acids for homeostasis, growth, and health maintenance. Furthermore, it is also important to state that in healthy individuals with a balanced diet, glutamine supplementation does not increase the efficacy of immune surveillance and/or prevent disease/sickness episodes.

On the contrary, during major and/or critical illness, sepsis, trauma, and post-surgery circumstances, patients suffer from chronic weakness and several nutritional limitations (e.g., state of unconsciousness, gastrointestinal disturbances, and/or chew related problems), which impair homeostasis, and are associated with poor clinical outcomes. Severe disturbances in amino acid metabolism and/or intermediary metabolism followed by skeletal muscle proteolysis are key characteristics of hypermetabolic/hypercatabolic states [167]. During hypercatabolism, some non-essential amino acids, including glutamine, become conditionally essential. As previously mentioned, glutamine is critical for cell homeostasis, and cells cannot survive and/or proliferate in an environment where glutamine is lacking. Therefore, the administration of non-synthetic amino acid supplements, such as glutamine, has been a research target in in the last many years and is currently indicated for hypercatabolic and/or ill patients. However, the efficacy of glutamine supplementation is frequently questioned due to confusing and controversial results [150,168,169].

**Figure 4:**
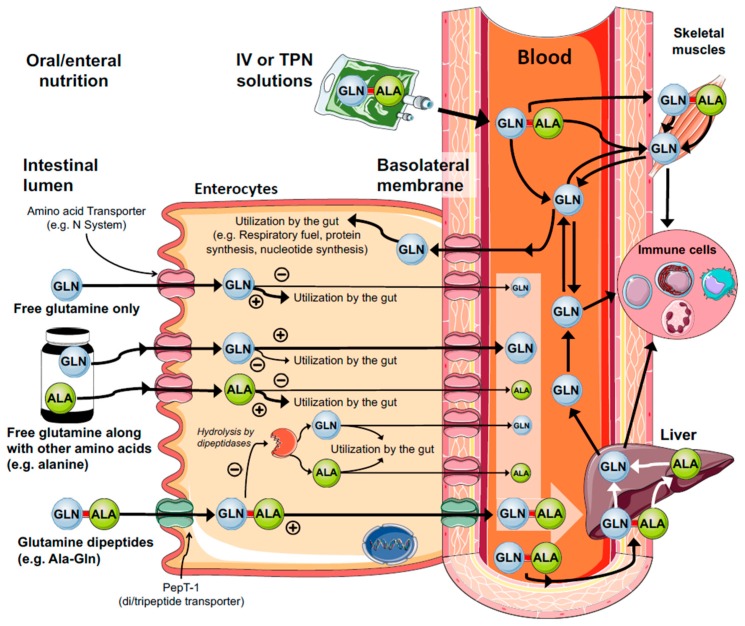
Mechanisms of enteral and parenteral glutamine (GLN) supply. Glutamine is an important substrate for rapidly dividing cells, such as enterocytes. This is a major site of glutamine consumption obtained from both exogenous/diet (luminal membrane) and/or endogenous glutamine synthesis (basolateral membrane). Free glutamine supplementation is mainly metabolized in the gut and poorly contribute to glutaminemia and tissue stores. On the other hand, glutamine dipeptides (e.g., Ala-Gln, Gly-Gln, Arg-Gln) escape from the gut metabolization and quickly supply glutamine to the plasma and target tissues. This effect is mainly attributed to the oligopeptide transporter 1 (Pept-1) located in the luminal membrane of the enterocytes.

Glutamine is usually administrated by utilizing its free form (also known as an isolated amino acid), or bond with another amino acid, also known as the dipeptide form (Figure 4). Several glutamine dipeptides with potential recovery health benefits have been described, such as l-glycyl-l-glutamine (Gly-Gln) and l-arginyl-l-glutamine (Arg-Gln); however, the most well-known is possibly l-alanyl-l-glutamine (Ala-Gln) [170]. Given parenterally, many clinical and experimental studies and appropriate systematic reviews [168] have concluded that glutamine dipeptides can reduce the rate of infectious complications [171,172,173,174], length of hospital stay [9,175], and mortality of critically ill patients [10,176,177]. The choice for free glutamine or glutamine dipeptides largely depends on the patient’s catabolic circumstance and/or the most suitable route of administration (e.g., enteral and parenteral nutrition). For instance, in patients receiving total parenteral nutrition (TPN), glutamine dipeptides offer several advantages, such as stability during sterilization, prolonged storage, and high range of solubility (154 g/L H_2_O at 20 °C, 568 g/L H_2_O at 20 °C, respectively) when compared to free glutamine (36 g/L H_2_O at 20 °C) [170]. Moreover, free glutamine is usually commercially available as a crystalline amino acid powder and can be diluted into commercially available TPN solutions, however, this procedure requires daily preparations at a controlled temperature (i.e., 4 °C), aseptic conditions followed by sterilization through specific membrane filtration, and the concentration should not exceed 1–2%. This is particularly important because, at a low concentration, TPN solutions will increase the patient’s fluid intake to meet the daily glutamine recommendation, however, this cannot be feasible for fluid restricted patients.

In both oral/enteral or parenteral nutrition, the typical glutamine daily administration (free and dipeptide forms) may vary from a fixed dose of 20–35 g/24 h to an adjusted dose of <1.0 g (usually 0.3 g–0.5 g) per kg of body weight [168]. As any other nutrient or medication administrated directly into the bloodstream when free or dipeptide forms of glutamine are given parenterally, the increase in plasma glutamine is superior when compared to oral/enteral feeds [178]. However, it should be noted that although parenteral routes can secure nutrient delivery to target tissues, it is always an invasive route and may increase the risk of infections per se. It is strongly recommended that the decision to use parenteral solutions must be based on several nutritional parameters, such as poor nutritional status, dramatic reduction of body weight and body mass index, low plasma albumin, and/or severe loss of nitrogen and tissue function.

For individuals with regular enteral feeds at home or hospitals, and also elite athletes where glutamine supplementation is eventually recommended, oral or enteral routes are always more physiological. Furthermore, enteral solutions stimulate the intestinal cells to produce other intermediary amino acid derivatives important for immunological functions, and also compromised in hypercatabolic patients, such as arginine and its downstream metabolites (e.g., ornithine and citrulline) [179]. Experimental studies in animal models and humans have shown an increase in glutaminemia between ±30 to ±120 min after oral/enteral free glutamine [50] or Ala-Gln supplementation [50,177]. However, the peak concentration and the area under the curve promoted by Ala-Gln tend to be superior when compared to free glutamine supply. This effect is largely due to the expression of the human oligopeptide transporter 1 (Pept-1) located in the luminal microvilli membrane of the enterocytes [180], and in a lesser extent, through paracellular mechanisms and cell-penetrating peptides’ translocation [181,182] (Figure 4). Pept-1 is a high capacity, low-affinity proton-coupled cotransporter of diverse di/tripeptides, which include the glutamine dipeptides. Pept-1 is considered the main route of protein absorption in mammals’ intestines since the protein is able to transport about 400 dipeptides and 8000 tripeptides derived from the 20 L-α amino acids [180]. As a result, free glutamine and/or alanine deriving from enteral Ala-Gln administration can be released into the bloodstream, thus making the amino acids available to target tissues, including the liver [183], immune system [39], kidneys [184] and skeletal muscles [53] (Figure 4). Interestingly, the effects promoted by Ala-Gln are also mediated by the presence of the amino acid, alanine, in the peptide formulation. Oral free glutamine along with free alanine promoted similar metabolic, antioxidant, and immunological effects when compared to Ala-Gln supplementation in in vivo animal models submitted to infection [4,39], and exhaustive aerobic [30,45,53] and resistance physical exercise [29,162]. Importantly, in all of these experiments, the supplemented groups received isonitrogenous and isocaloric solutions, i.e., both containing 13.46 g of glutamine/100 mL and 8.20 g of alanine/100 mL. Although the precise mechanisms are still unknown, it is clear that both amino acids work in parallel, especially in absorptive cells (Figure 4). For instance, alanine is rapidly metabolized via alanine aminotransferase to pyruvate, with concomitant production of glutamate from 2-oxoglutarate, which contribute to antioxidant defence mediated by GSH. Although other free amino acid combinations need to be tested, these important discoveries may lead to the design of new formulations for specific hypercatabolic patients.

Oral/enteral or parenteral doses of glutamine supplementation have been tested in hundreds of studies in both animal models and humans, and if offered as a single nutrient supplementation (not combined with other additives), can be considered safe. In addition, there is no scientific evidence demonstrating that glutamine supplementation can suppress and/or inhibit permanently its endogenous production or de novo synthesis. However, as any other amino acid offered in excessive doses, it can promote hyperaminoacidemia and result in poor clinical outcomes. It is considered not to be the best practice to provide glutamine supplementation to patients without a proper evaluation that is supported by a nutritional assessment and biochemical laboratory tests.

Glutamine metabolism and supplementation in cancer has also raised many concerns among the scientific community, and deserve some comments. It is well established that cancer cells are extremely dependent of glutamine metabolism and availability, however, the role played by glutamine in cancer/tumours cells in vivo is still controversial, and thus the effects of the supplementation. Cancer cells take advantage of aerobic glycolysis (also known as the Warburg effect), and therefore glucose to maintain the supraphysiological survival and growth [185,186]. On the other hand, there is an increasing evidence of the role of oncogenes and tumour suppressors in the regulation of nutrient metabolism [187]. Aberrant mutations in these genes lead to altered nutrient metabolism, and can significantly contribute to the development and/or progression of cancer cells [186]. For instance, glucose, glutamine, lipids, and acetate can be utilized as carbon and energy sources [25]. To increase the level of complexity, the nutrient sources may vary among different types of cancer and/or tumour cells and are highly heterogeneous [25,187]. For example, lung cancer cell lines are highly dependent of glutamine supply in vitro, however, in vivo experiments demonstrate that glucose is the preferred source of carbons supplied to the Krebs cycle, through the action of pyruvate carboxylase [188], with little changes in glutamine consumption [187]. Human and mouse gliomas exhibit high rates of glucose catabolism, and use glucose to synthesize glutamine through glutamate-ammonia ligase (GLUL), which in turn promotes nucleotide biosynthesis via the pentose phosphate pathway independently of circulating glutamine [189,190]. Conversely, prostate cancer cell lines exhibit aberrant intracellular lipid metabolism [191], and an increased gene expression of glutaminolitic enzymes and glutamine transporters, thereby stimulating cell growth via glutamine uptake [192].

The variances in cancer cells’ nutrient metabolism suggest that different nutritional approaches should be taken into consideration. However, studies have also targeted whether a glutamine exogenous supply may attenuate the side effects promoted by chemotherapy and radiation in cancer patients [193]. In a systematic review, Sayles, et al. [194] reported that in 11 of 15 studies, oral glutamine supplementation (dose range: 30 g/day in 3 divided doses, or 7.5–24 g/day) significantly reduced the grade of mucositis (common in 90% of head/neck cancer patients) [193] and/or attenuated weight loss in cancer patients. In a double-blind, placebo-controlled, randomized trial, colorectal cancer patients undergoing chemotherapy were supplemented with 18 g/day of glutamine (five days before and during the treatment). Glutamine treatment reduced the side-effects induced by chemotherapy, such as intestinal absorption and permeability, diarrhea, and gut mucositis [195]. As a whole, it is important to highlight that glutamine supplementation for cancer patients may also fuel certain types of cancer cells, and have a negative impact on health. However, considering the strong ability of metabolic switching of cancer cells, glucose and/or lipids can also induce similar effects, and therefore it would be difficult for a human to survive and maintain immunity and/or immune surveillance without these nutrients. As mentioned previously, a proper and possibly individualized patient evaluation is required to determine the suitability of glutamine supplementation.

Low plasma glutamine level (hypoglutaminemia) is usually used as a parameter to indicate the need for a glutamine exogenous supply. However, the correlation between the concentration of glutamine in plasma and tissue vary significantly between hypercatabolic patients, and therefore among studies [150]. For instance, muscle glutamine was dramatically reduced in abdominal surgery patients, but no changes were detected in plasma [196]. In critically ill patients, however, there is a profound drop in muscle glutamine, but a variable reduction in plasma [150]. Other important glutamine sites, such as the gut and the liver, may show a concomitant plasma and tissue glutamine reduction, or even an inverse relationship during major illness [86,197,198]. These findings are also in agreement with data obtained in rats [29,45,183] and mice [4,199] submitted to infection and exhaustive exercise.

The variations between plasma and tissue glutamine concentration are due to the fact that only a small fraction of the total body free glutamine is in plasma. To add to the confusion, it is known that cells of the immune system, such as lymphocytes maintained in a low glutamine availability (similar to low plasma glutamine concentration, e.g., ±400 μM), still proliferate, when compared to resting values (e.g., 600 μM) [4,7,90]. The rate of macrophages’ phagocytosis and cytokine production by diverse peripheral blood mononuclear cells (PBMCs) is also dependent on glutamine availability, with decreasing rates at ±<600 μM of glutamine [21,200]. Thus, for some catabolic/hypercatabolic patients, the changes in glutamine concentration, especially in plasma, will not necessarily affect and suppress immune functions, and possibly no significant changes might be observed in data obtained from immune parameters and function, and mortality risk predictors, such as APACHE II or SAPS III. Taken together, hypoglutaminemia can only be interpreted as an independent variable of mortality and/or poor clinical outcome [10,93,168,169,174,201]. More in-depth studies are required to explore the specific relationship between dramatic changes in plasma glutamine and outcomes in critically ill patients. Currently, the decision of glutamine supplementation should be based in a set of immune-inflammatory parameters allied with appropriate nutritional assessment, and eventually, risk predictors. In addition, glutamine supplementation studies cannot be judged on trials where only the very sickest patients (i.e., with two or more organ failures) were eligible for this nutritional intervention, and in these situations, supraphysiologic doses are not an appropriate nutritional solution.

## 7. Conclusions and Future Perspectives

Immune cells largely depend on glutamine availability to survive, proliferate, and function, and ultimately defend our body against pathogens. During catabolic/hypercatabolic circumstances, the demand for glutamine increases dramatically, a fact that may lead to a glutamine deprivation and severe impairment of the immune function. However, low glutamine availability is not observed in every catabolic/ill or critically ill patient, and thus not all individuals will benefit from glutamine supplementation. It is important to consider that like glycaemia, plasma glutamine and the inter-tissue metabolic flux is maintained at constant levels even during high catabolism by key organs, such as the gut, liver, and skeletal muscles. Not surprisingly, hypoglutaminemia conditions and severity vary significantly between human and animal studies, and by itself do not provide a rational argument for glutamine exogenous supply.

For some catabolic situations, and/or where there is a shortage of glutamine obtained from the diet, amino acid supplementation might be required. In this regard, the immune properties of glutamine supplementation have been extensively studied and new questions and perspectives are formulated. For instance, studies should determine the frequency of nutritional intervention, optimal doses associated with the disease or stress situation, and the concomitant administration with other amino acids or dipeptide combinations. In addition, the evolving metabolomics era has the potential to improve our understanding of the complex regulation of glutamine metabolism, identifying new metabolites (e.g., Itaconate in macrophages) critical for cell function, thus going beyond the concept of “the fuel for the immune system”.

## Figures and Tables

**Table 1:** Total protein, glutamine, glutamate, and leucine (g/100g food) content in some animal and vegetable foods using the gene sequencing method (adapted from [166]).

g/100g Food	Beef 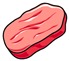	Skim Milk 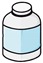	White Rice 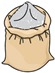	Corn 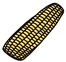	Tofu 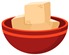	Egg 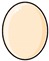
Total protein	25.9	3.4	2.7	2.5	6.6	12.6
Glutamine	1.2	0.3	0.3	0.4	0.6	0.6
Glutamate	2.7	0.4	0.2	0.05	0.7	1.0
Leucine	2.2	0.4	0.2	0.4	0.5	0.9
